# Prior use of antibiotics and immunosuppression are risk factors for fracture-related infection during the COVID-19 pandemic period: a Brazilian prospective cohort study

**DOI:** 10.1186/s12891-022-05493-5

**Published:** 2022-06-04

**Authors:** Eduardo Cezar Santos, Stefânia Prebianchi, Ingrid Nayara Santos, Mariana Neri Kurihara, Adriana Dell’Aquila, Carlos Finelli, Fernando Baldy dos Reis, Mauro José Salles

**Affiliations:** 1 Department of Orthopedic, Escola Paulista de Medicina, Federal University of São Paulo – UNIFESP, São Paulo, Brazil; 2 Laboratório Especial de Microbiologia Clínica (LEMC), Universidade Federal de São Paulo, Escola Paulista de Medicina, São Paulo, SP Brazil; 3 Department of Internal Medicine, Division of infectious Diseases, Escola Paulista de Medicina, Federal University of São Paulo, São Paulo, Brazil; 4 Department of Internal Medicine, Division of Infectious Diseases, Faculdade de Ciências Médicas da Santa Casa de São Paulo, São Paulo, Brazil

**Keywords:** Fracture-related infection, Elderly, COVID-19 pandemic, Risk factors, Gram-negative bacteria, Multidrug-resistance, Prior use of antibiotic, Cancer

## Abstract

**Background:**

Little is known about the role of COVID-19 pandemic period on the epidemiology of fracture-related infection (FRI). The present study summarizes the changes in the prevalence, microbiology, and risk factors of FRI during this period.

**Methods:**

A prospective single-center cohort study assessed in the setting of COVID-19 pandemic (2020–2021), clinical, microbiological aspects, and independent risk factors (RF) of FRI. RFs were estimated by bivariate and multivariable analyses using prevalence ratio (PR) with significance at *P* < 0.05. Kaplan–Meier analysis was performed to evaluate treatment outcomes.

**Results:**

Overall, 132 patients were analyzed, with patients with age over 65 years accounting 65.1%. FRI was diagnosed in 21(15.9%) patients. Independent RFs for FRI were recent and preoperative use of systemic antibiotics (PR: 7.0, 95% confidence interval (95% CI): 2.2 – 22.4, *p* = 0.001) and cancer (PR: 9.8, 95% CI: 2.0 – 48.8, *p* = 0.005). Cultures yielded Gram-negative bacteria in 77.8%, 33.3% were MDR.

**Conclusions:**

We found higher rates of FRI, predominating in the elderly with closed femoral fractures during the COVID-19 pandemic. Prior use of antibiotics and immunosuppression conditions were independent factor for FRI. Our outcomes provide evidence to avoid the empirical use of antibiotics prior to surgery for fracture stabilization.

**Supplementary Information:**

The online version contains supplementary material available at 10.1186/s12891-022-05493-5.

## Introduction

Fracture-related infection (FRI) remains one of the main concerns among victims of orthopedic trauma due to the higher frequency of unfavorable outcome, especially among patients with open fractures and extensive soft tissue injuries [1, 2]. The infection rate after open reduction and internal fixation (ORIF) ranges from 0.4 to 16.1%, in open fractures, but it may be higher depending on many factors including the degree tissue damage and contamination, and patient’s underlying chronic medical condition [3]. Recent studies show that the average cost per FRI-patient can reach US$108,000 dollars, with a cure rate ranging between 70 and 90% [4].

In the context of the COVID-19 pandemic period, surgical procedures for orthopedic fractures stabilization turned out to be a challenge to accomplish. Due to the overwhelming need to treat SARS-CoV-2 virus severe cases and the consequent readjustment of hospitalization flows, surgeries for open and closed fractures stabilization were frequently postponed, usually prolonging the length of hospital stay [5]. Moreover, with the social mobility lockdown during this period of time, there seems to have been a decrease in road traffic accidents and the consequent increase in domestic trauma, such as hip and femur closed fractures in the elderly [6]. Additionally, it is known that SARS-CoV-2 virus infection triggers a pro-inflammatory cytokines storm that in conjunction with the inflammatory process following bone fractures, is likely to influence the morbidity and mortality among these patients [7]. This risk could be mitigated among those undergoing elective surgeries in which a minimum period of 6 weeks was recommended between the COVID-19 diagnosis to the surgical procedure [8]. Nevertheless, for patients undergoing fracture correction in emergencies, co-infection with SARS-CoV-2 virus may be difficult to be ruled out and prevent [9].

Despite the well-known poor outcome of infection following orthopedic fractures on the quality of life of individuals and the higher impact on the socioeconomic status of public or private health care systems, the role of COVID-19 pandemic period (2020–2021) on the epidemiology and management of FRI has yet to be well understood [10]. Therefore, the present study aims at describing the changing epidemiology of patients treated for closed and open fracture stabilization during the SARS-COV-2 pandemic period, the FRI rates with at least one-year of follow up, the microbiological profile and to analyze independent risk factors for FRI.

## Materials and methods

### Study design

This is a prospective observational single-center cohort study conducted during the 2020–2021 COVID-19 pandemic (March 2020 to March 2021), carried out at a specialized orthopedic trauma center in São Paulo/Brazil, among patients undergoing any surgical treatment of open and closed fractures, in which the diagnosis of FRI had been performed. The primary purpose was to assess in the setting of COVID-19 pandemic period, the outcome of patients treated for fracture stabilization, including the prevalence of fracture-related infection (FRI), the microbiological epidemiology and to analyze independent risk factors for FRI. The study was previously approved, with the waiver of the free and informed consent form, by the Ethics and Research Committee of the Federal University of São Paulo (UNIFESP) under the number: 0846P/2021.

Inclusion criteria were patients aged 18 years old or over, trauma victims with open and/or closed fractures who underwent any type of surgery for bone fracture stabilization using plate and screw, intramedullary nail (IMN) and Kirschner wires. Patients undergoing arthroplasties or spine instrumentation, severe cases likely to progressed to death within the first 7 days of hospitalization, and patients unable to undergo outpatient follow-up during the study period, and those who did not undergo any type of surgical intervention were excluded. Fracture-related infection (FRI) was defined according to criteria published by Metsemakers et al. [11]. Multidrug-resistant microorganism (MDR) was defined as the non-susceptibility of the identified pathogen to at least one antimicrobial agent from 3 or more different antimicrobial classes [12, 13].

### Data collection and variables analyzed

Data were prospectively collected and recorded in data collection forms by the specialists in the musculoskeletal infection group (ECS, SP**)** based on information from electronic medical records filled out during hospitalization and outpatient follow-up for up to one year after index surgery. The variables were divided as follows: variables related to the patient (1), related to the surgical and perioperative procedure (2). The variables related to the patient were (1): demographics, comorbidities, alcoholism, smoking, ASA (American Society of Anesthesiologists) physical status classification, Charlson comorbidity index, previous use of antimicrobials in the last three months and previous orthopedic infections. Surgical and perioperative procedure were (2): location and classification of the fracture, type of fracture, time between fracture and surgical treatment, prophylactic antimicrobial therapy, COVID-19 symptomatic infection, time of antimicrobial therapy, concomitant infection in the same period of hospitalization, presence of postoperative hematoma, presence of sepsis at the time of infectious diagnosis and early or late infection, result of blood culture sample and microbiological profile.

### Microbiological analysis

During the surgical procedure for diagnosis and treatment of FRI, between 3 to 5 samples of peri-implant tissue and bone were collected aseptically, placed in properly labeled sterile containers and sent to the microbiology and histopathology laboratory. In the laboratory, tissue samples were homogenized in 3 ml of Brain Heart Infusion (BHI) agar for 1 min and inoculated on aerobic blood agar, chocolate agar and anaerobic blood agar in a flask at 35ºC, and also in Thioglycolate (TG) medium (BD Diagnostic Systems, Sparks, MD). Blood agar and chocolate agar plates were then incubated at 35–37 °C for 5 days for aerobic cultures and 14 days for anaerobic cultures. TG broth was incubated for 14 days, and in case of bacterial growth (turbidity), the liquid was seeded on blood agar plates (aerobic and anaerobic cultures). Microbiological methods for synovial fluids were similar, inoculating 0.1 mL on agar and liquid broth plates and evaluating aerobically and anaerobically. Isolated bacterial colonies were identified by matrix-assisted laser ionization-desorption-time-of-flight (MALDI-TOF MS) (Bruker Daltonics, Germany). The sensitivity profile was determined for all identified strains according to the prevailing standards of the Clinical Laboratory Standards Institute (CLSI) (Standardization of Antimicrobial Disk Diffusion Susceptibility Testing: Approved Standard—8th Edition, 2010, Vol. 23 No 1) [14]. Low virulence microorganisms (*Staphylococcus epidermidis*) were considered pathogenic when the organism was found in at least 2 different tissue culture samples [14, 15].

### Statistical analysis

Data analysis included mean, median, standard deviation, interquartile range, variance for continuous variables and frequencies and proportion for categorical variables. The association between two categorical variables was performed using Pearson's Chi-square test or Fisher's exact test for categorical variables. The Kaplan–Meier test and the log-rank test were used to assess the influence of the variables of interest on survival time, that is, the time until infection occurs. A Cox regression model was fitted to identify factors related to time to infection. Risk estimates were calculated on the variables associated with risk factors for FRI and reported as a prevalence ratio (PR) with respect to the 95% confidence interval (CI). Variables with *p* ≤ 0.25 were selected for multivariable analysis. All results were considered significant for a probability of significance lower than 5% (*p* < 0.05), therefore having at least 95% confidence in the conclusions presented.

## Results

**Fig. 1:**
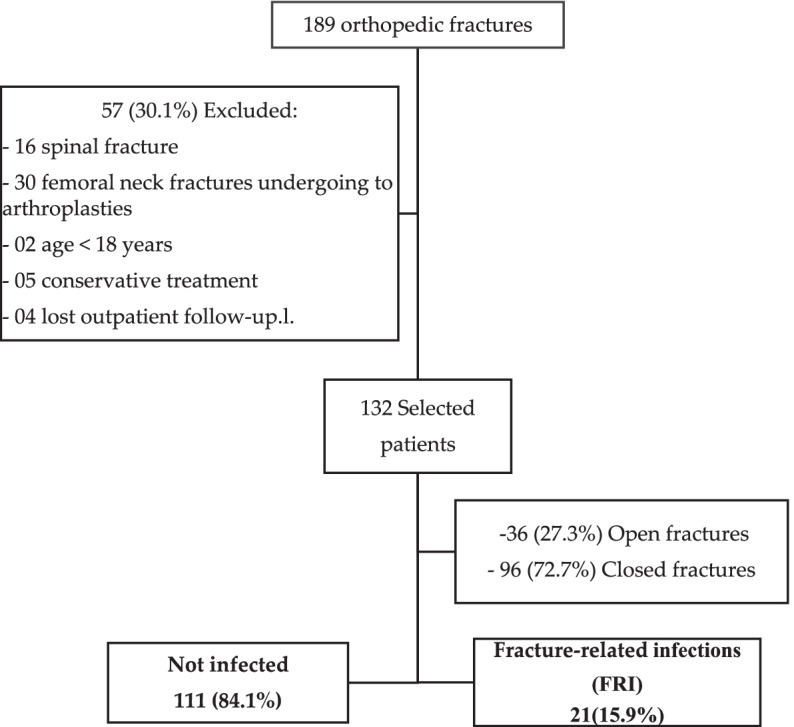
Diagram of patients involved in the study

Overall, 189 patients with orthopedic fractures were evaluated for inclusion in the study. Of these, 57 (30.1%) were excluded: spine fracture (16), femoral neck fracture undergoing arthroplasty (30), two patients excluded for age under 18 years, 05 did not undergo surgical correction and 04 patients lost prospective outpatient follow-up. A total of 132 patients who underwent surgical correction of fractures were selected for analysis and prospective followed, of which male sex was 75%, with mean age of 50.4 years (SD ± 22.9 years). Patients with age over 65 years accounted 65.1%. Charlson comorbidity index with a low score (up to 4 points) was diagnosed in 80.9% of patients. Fractures due to road traffic accidents (motorcycle/car) and falls from standing height occurred in 42.4% and 30.3%, respectively. Lower limbs fractures accounted for the majority of cases (80.3%). Closed and open fractures were 72.7% (96/132) and 27.3% (36/132), respectively. Out of 36 open fractures, 69.4% were classified as Gustilo-Anderson III or higher. Fracture-related infections (FRI) were diagnosed in 21 (15.9%) patients, of which 13 (9.8%) and 8 (6.1%) were open and closed fractures, respectively. (Fig. 1).

Temporary external fixator insertion for the first surgical fracture stabilization was placed in 20.6% of patients. Definitive surgical correction of the fracture within 72 h of the trauma occurred in 66.7%. Importantly, during the study period 3.8% (5/132) of patients evolved with symptomatic Covid-19 infection after surgery, requiring specific respiratory assistance in the intensive care unit. In addition, 3.8% (5/132) of patients described a recent preoperative use of systemic antibiotics for any cause. Demographic, pre- and post-surgical findings can be found in Table 1.

**Table 1:** Demographic and clinical characteristics associated with trauma and treatment of 132 patients evaluated

Variables	Results N(%)
**Gender**	
Male	99 (75)
**Age (years)**	
Mean ± Standard Deviation	50.4 ± 22.9
P_50_ (P_25_ – P_75_)	44.0 (31.0; 70.7)
**Age group**	
up to 21 years old	11 (8.3)
From 22 to 40 years old	44 (33.3)
From 41 to 60 years old	31 (23.5)
> 60 years old	46 (34.9)
**Comorbidities**	
Arterial Hypertension	30 (22.7)
Diabetes Mellitus	15 (11.3)
Renal KidneyDisease	6 (4.5)
Cancer	4 (3.0)
Others comorbidities	13 (9.8)
**Surgical risk classification ASA**^b^	
1	97 (73.5)
≥ 2	35 (16.5)
**Preoperative use of ATB**	
Yes	5 (3.8)
**Charlson Index** ^a^	
Up to 4	106 (80.9)
Over 4	25 (19.1)
**Time between Fracture and surgery (hours)**	
Up to 24 h	51 (38.7)
From 25 to 72 h	37 (28.0)
Over 72 h	44 (33.3%)
**Type of fracture**	
Open fracture	36 (27.3)
Closed fracture	96 (72.7)
**Gustilo Anderson Classification (G-A)**	
Type I-II	11 (30.6)
III A, B and C	25 (69.4)
**Type of implant used**	
Plate/Screw	58 (43.9)
intramedullary nail	74 (56.1)
**Nail at the proximal end of the femur**	
Yes	37 (50.0)
**Affected limbs**	
Lower	106 (80.3)
Upper	23 (17.4)
Lower / Upper	3 (2.3)
**External fixator** ^a^	
Yes	27 (20.6)
**Mechanism of injury**	
Motorcycle / car accident	56 (42.4)
Fall from one's own height	40 (30.3)
Fall from height	19 (14.4)
Others	17 (12.9)
**Infection by COVID**	
Yes	5 (3.8)

^a^—one undisclosed patient, ASA^b^—American Society of Anesthesiologists

The risk variables showing statistical significance of FRI in the univariate analysis were recent use of antibiotics in the preoperative period (*p* = 0.002), type of fracture (open vs. closed, *p* < 0.001), use external fixator (with vs. without, *p* = 0.015), osteosynthesis with plate and screw (*p* = 0.006), mechanism of injury (car accident vs others, *p* = 0.023), infection by COVID (*p* = 0.028) (Table 2).

**Table 2:** Risk factor for the infection outcome according to univariate analysis

Analyzed variable	Not Infected (*n* = 111)	Infected (*n* = 21)	*p-value*
**Demography**	N (%)	N (%)	
Male	84 (75.8)	15 (71.43)	0.680*
Age (average-years)	50.8	48.5	0.651**
P50(p_25_-p_75_)	44.0 (31.0; 72.0)	48.0 (30.5; 64.0)	
**Comorbidities**			
Habit of smoking	30 (27.0)	5 (23.8)	0.759
Alcoholism	29 (26.1)	4 (19.0)	0.492*
Arterial hypertension	23 (20.7)	7 (33.3)	0.255***
Cancer	3 (2.7)	2 (9.5)	0.179***
**Perioperative risk**			
Classification ASA^b^			
1	83 (74.7)	14 (66.6)	0.440*
≥ 2	28 (25.2)	7 (33.3)	
Charlson index^a^			
Up to 4	88 (79.2)	18 (85.7)	0.763***
Over 4	22 (19.8)	3 (14.2)	
**Associated with trauma**			
Mechanism of injury			
Fall from one's own height	38 (3.4)	2 (9.5)	
Motorbike/car accident	47 (42.3)	9 (42.8)	0.023***
Fall from height	4 (3.6)	3 (14.2)	
Open fracture	23 (20.7)	13(61.9)	< 0.001*
Closed fracture	88 (91.7)	8 (8.3)	
Gustilo-Anderson			
I	4 (3.6)	2 (9.5)	
II	4 (3.6)	1 (4.7)	0.688*
III A, B and C	15 (13.5)	10 (47.6)	
External fixator^a^	18 (16.2)	9 (42.8)	0.015***
Low Limber fracture	88 (79.2)	18 (85.7)	0.861***
Time interval Fracture-surgery(h)	87.8 ± 119.8	93.3 ± 118,8	0.847**
Average p_50_ (p_25_ – p_75_)	48 (24; 96)	24 (12; 121.5)	
**Perioperative data**			
Type of osteosynthesis			
Plate and screw	43 (38.7)	15 (71.4)	0.006*
Locked intramedullary nail (IMN)	68 (61.2)	6 (28.5)	
Recent preoperative use of ATB	1 (0.9)	4 (19.0)	0.002***
Infection by covid	2 (1.8)	3 (14.2)	0,028***

The probability of significance refers to the Chi-square test (^*^), Student's t test (^**^), Fisher's exact test (^***^), ^a^—one undisclosed patient, *ASA*^b^ American Society of Anesthesiologists, *ATB* Antibiotic

**Fig. 2:**
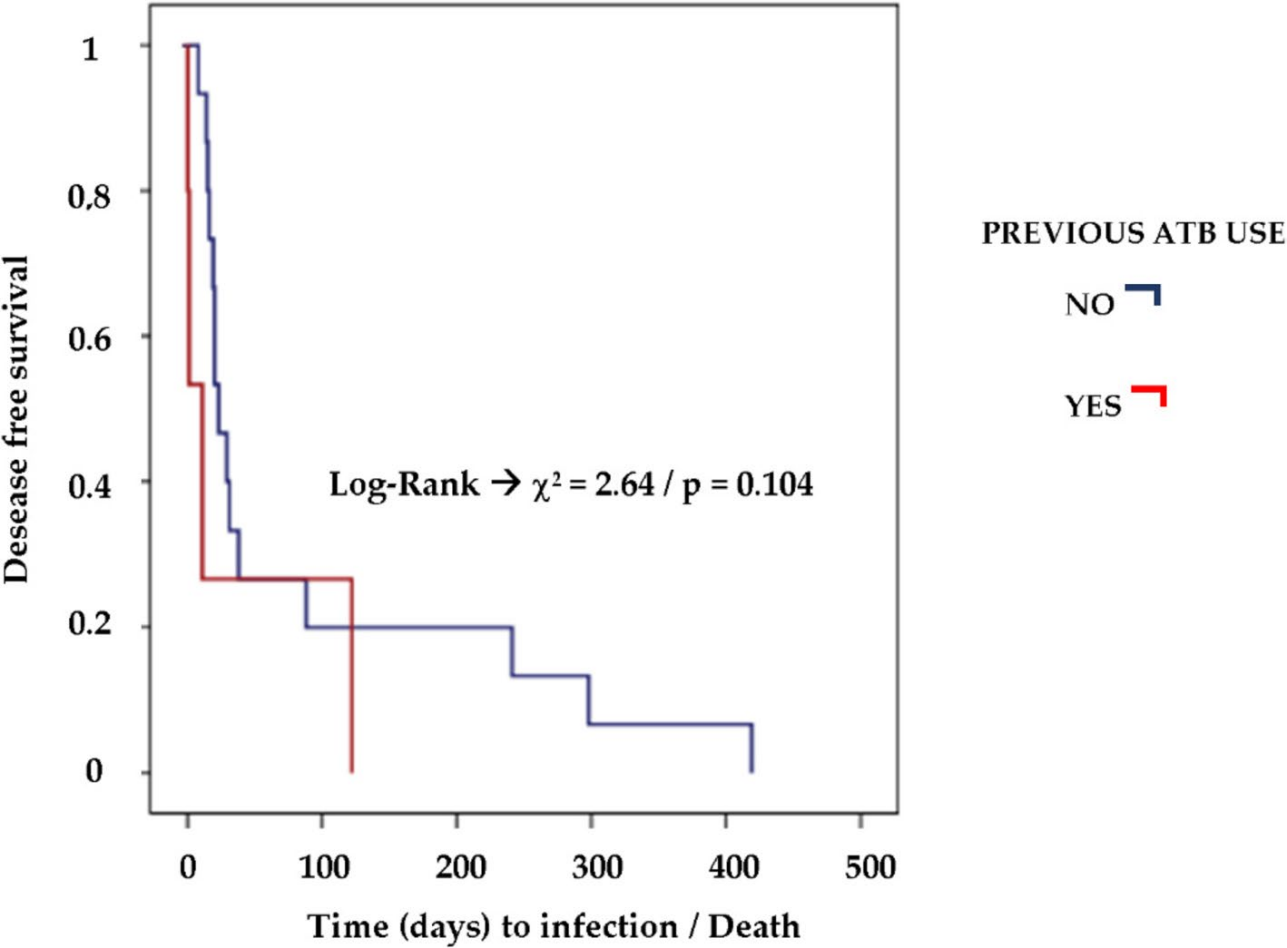
Kaplan–Meier survival curve for infection considering recent preoperative use of ATB. DATABASE: 130 cases (*No* 125 cases and *Yes* 5 cases); ATB Antibiotic

However, in the multivariable analysis, only recent and preoperative use of systemic antibiotics and the presence of cancer were independent risk factors for FRI (Table 3). Indeed, among those with recent antibiotic intake, the incidence of FRI was 7 times higher (CI 2.2; 22.4) than in the group that did not use antibiotics. While the presence of cancer increased the risk for FRI by approximately 9.8 times (CI 2; 48.8). These two variables were found mainly in elderly patients. In the survival analysis to identify independent risk factors related to time to diagnosis of FRI and death, prior use of antibiotic, habit of smoking and open fractures influenced the time to FRI or death with statistical significance. (Table S1 and Fig. 2).

**Table 3:** Risk factors independently associated to infection outcome–multivariable analysis

Analyzed Variable	PR (95% IC)	*p-value*
Recent preoperative use of ATB	7.0 (2,2; 22.4)	0.001
Cancer	9.8 (2; 48.8)	0.005

Significance probabilities in multivariable analysis refers to Poisson regression, PR prevalence ratio, ATB – antibiotics

Regarding microbiological findings among 21 FRI patients, tissue cultures were negative in six (28.5%), and polymicrobial in 4 (19.2%). The most frequent isolated microorganisms were *Klebsiella pneumoniae* (27.8%) and other Enterobacteriaceae (77.7%). Fewer Gram-positive cocci (22.2%) and no *S. aureus* were identified. Overall, seven patients (33.3%) were infected by multi-resistant microorganisms (Table S2).

## Discussion

There are few prospective studies carried out during the COVID-19 pandemic period aiming to describe the current changes in the epidemiology and risk factors of FRI [16]. In our study the overall FRI rate was 15.9%, which is high compared to the 11.6% described by Kamat et al. [17] and similar to the 14.9% described by Singh et al. [18].

Rather than road traffic accidents, domestic trauma seemed to have stood out during the pandemic period especially falls from any height involving the elderly due to the long period of social distancing and domestic confinement [16, 19]. On top of that, the presence of comorbidities and the severe co-infection by SARS-CoV-2 virus also became important risk factors for FRI in this population, according to the multicenter study by McDonald et al. [20]. This study compared injuries and trauma mechanisms before and during the pandemic, depicting a reduction in the number of fractures in general, but an increase of over 30% of hip fractures. Additionally, the author showed an increase in the age range of the population. The present study also identified a similar clinical and epidemiological trend related to patient’s demographics, comorbidities, and characteristics of trauma. Moreover, despite having few severe cases (five) of COVID-19 infection, it proved to be a significant factor for FRI in the univariate analysis and likely influenced towards the higher rates of FRI. We also hypothesized that this shift may have been due to the restrictive measures implemented during the lockdown to contain the virus progress. Besides, the SARS-CoV-2 virus infection severity and consequently increased in the length of hospital stay justifies the frequently delay for definitive surgical fracture stabilization which some of our patients experienced during this pandemic period. Among many modified and non-modified factors, delayed definitive fracture stabilization following closed trauma is thought to impact in the rates of FRI in the elderly [21].

An important find in the present study was the role of recent use of preoperative antibiotics which was a strong and independent association with FRI. Patients with a history of previous used antibiotics had 7 times higher risk of FRI than patients who had not. This data is very relevant due to few previous reports in the literature. In their series of 502 patients, Declerq et al. [22] showed that the use of an antimicrobial regimen to treat an infection unrelated to the fracture was statistically significant for the occurrence of FRI. However, there were unclear reasons for this association. Although not analyzed in the study, we argue that an over prescription of empirical antibiotic therapy is likely to have occurred during the entire COVID-19 pandemic period, including within the orthopaedic inward of our tertiary hospital where a higher number of elderly patients with closed fractures underwent orthopaedic surgery. Recent published multicenter studies corroborate this argument, showing an overuse of empirical antibiotic therapy among hospitalized patients [23]. Besides, inappropriate prescription of antibiotics increases the selective pressure on patient`s bacterial microbiota impacting on the development of drug-resistant bacterial infections [24]. Multidrug-resistant Gram-negative bacteria were the causative agents of 33% of FRI in the present study.

Comorbidities in the elderly population is common, including diabetes, hypertension, chronic heart diseases and cancer which impairs the immune response. In our study the presence of any type of cancer increased the risk for FRI by approximately 9.8 times. Conversely, in a case series study of 480 patients, Metsemakers et al. [25] observed that the healthier patients without immune-depressive comorbidities evolved with lower infection rate.

The role of high-energy open fractures in the pathogenesis of FRI is well investigated [26, 27], while in the present study it was independently associated with time to diagnosis of FRI. In fact, more than two-thirds of FRI cases comprised open fractures. In the setting of public healthcare system in developing countries, the management of patients with high energy open fractures of the lower limb due to road traffic accidents may be hampered, increasing the likelihood of FRI [28–30]. Nevertheless, we are in accordance with Morgenstern et al.[31] emphasizing the importance of having a holistic and multidisciplinary approach in the prevention of FRI following trauma. In this regard patient`s characteristics including common habits are taken into consideration. Several studies associate smoking with poor surgical treatment outcomes for fractures, although the evidence is still contradictory [32, 33]. In our series, smoking was independently influenced the time to diagnosis of FRI.

The study has limitations for being carried out in a single center of a large tertiary teaching hospital offering special orthopaedic care to a regional population located in a major city in a developing country, which limits the global comparison of the data. Besides, the overall number of FRI was low that may have biased our results, although multivariable analyses were performed to identify independent factors related to time and diagnosis of infection. Importantly, another limitation is related to patient`s selection bias as we excluded those with extreme ill conditions with greater immunological susceptibility to infections and likely to progress to death within the first 7 days of hospitalization. Nevertheless, we included in the analysis patients with severe comorbidities such as neoplasia and those with extreme advanced ages. A challenging factor was carrying out the study during the lockdown period, which made it difficult to diagnose and treat FRI. However, the patients were followed prospectively, for a period of at least 12 months, and most importantly the definitions of FRI used were standardized and well-defined according to Metsemakers et al. [12], decreasing the chance of bias. Moreover, the robust statistical analysis of the data contributes relevant information for planning higher impact study designs.

## Conclusion

In conclusion, in this single-center prospective study we found higher rates of FRI, predominating in the elderly with closed lower limb fractures during the COVID-19 pandemic period. Prior use of antibiotics and presence of cancer were independent factor for FRI. The majority of infection were due Gram-negative bacteria with higher rates of MDR. Our outcomes provide evidence to avoid the empirical use of antibiotics prior to surgery for fracture stabilization. Further analysis during the lockdown period may be needed to confirm these results.

## Supplementary Information

**Additional file 1: Table S1.** Assessment of the influence of variables in time until infection diagnosis. **Table S2.** Frequency of microorganisms isolated in tissue cultures.

## Data Availability

Epm, Ortoinfecto (2022), “MUSCULOSKELETAL INFECTION”, Mendeley Data, V1, https://doi.org/10.17632/grxjspbf29.1.

## Abbreviations

ASA: American Society of Anesthesiologists
ATB: Antibiotic
BHI: Brain-heart infusion
CI: Confidence interval
COVID: Coronavirus infection diseases
CLSI: Clinical Laboratory Standards Institute
DAIR: (Debridement, antibiotics, and implant retention)
FRI: Fracture relationed infection
G-A: Gustillo & Anderson
IMN: Intramedullary Nail
MALDI-TOF MS: Matrix-assisted laser ionization-desorption-time-of-flight
MDR: Multidrug-resistant
OR: Odds ratio
PR: Prevalence Ratio
RF: Risk factors
SD: Standard deviation
TG: Thioglycolate
UNIFESP: Federal University of São Paulo
