# Supplementary material for: Prior use of antibiotics and immunosuppression are risk factors for fracture-related infection during the COVID-19 pandemic period: a Brazilian prospective cohort study
Source: BMC Musculoskelet Disord. 2022 Jun 4;23:535. doi: 10.1186/s12891-022-05493-5 (PMC9166157; doi:10.1186/s12891-022-05493-5)
Supplement: Supplementary file 1 — Additional file 1: Table S1. Assessment of the influence of variables in time until infection diagnosis. Table S2. Frequency of microorganisms isolated in tissue cultures. [file 12891_2022_5493_MOESM1_ESM.docx]

**Supplementary Materials**

**Abbreviations:**

ASA: American Society of Anesthesiologists; ATB: Antibiotic; BHI: Brain-heart infusion; CI: Confidence interval; COVID: Coronavirus infection diseases; CLSI: Clinical Laboratory Standards Institute; DAIR: (Debridement, antibiotics, and implant retention); FRI: Fracture relationed infection; G-A: Gustillo & Anderson; IMN: Intramedullary Nail; MALDI-TOF MS: matrix-assisted laser ionization-desorption-time-of-flight; MDR: Multidrug-resistant; OR: Odds ratio; PR: Prevalence Ratio; RF: Risk factors; SD: Standard deviation; TG: Thioglycolate; UNIFESP: Federal University of São Paulo.

**Table S1 -** Assessment of the influence of variables in time until infection diagnosis.

| **Analyzed Variable** | **Univariate Analysis (*p-value*)** |  | **Multivariate**  **analysis**  **RR (95% IC)  *p-value*** | |
| --- | --- | --- | --- | --- |
| Previous ATB use | 0.104 |  | 3.7 (1.0;13.4) | 0.043 |
| Habit of Smoking | 0.036 |  | 4.2 (1.1;16.1) | 0.035 |
| Open Fracture | 0.098 |  | 3.1 (1.1; 8.9) | 0.034 |

**Note:** Significance probabilities in multivariate analysis refer to Cox regression. RR 🡪 relative risk; ATB – antibiotic.

**Table S2:** Frequency of microorganisms isolated in tissue cultures.

| **Microorganism** | **N** | **%** |
| --- | --- | --- |
| *Klebsiela pneumoniae* | 5 | 27.8 |
| *Acinetobacter baumanni* | 2 | 11.1 |
| *Escherichia coli* | 2 | 11.1 |
| *Staphylococcus epidermidis* | 2 | 11.1 |
| *Pseudomonas aeruginosa* | 2 | 11.1 |
| *Enterobacter cloacae* | 1 | 5.5 |
| *Enterococcus spp* | 1 | 5.5 |
| *Staphylococcus intermedius* | 1 | 5.5 |
| *Stenotrophomonas maltophilia* | 1 | 5.5 |
| *Providencia spp* | 1 | 5.5 |
